# *SPARCL1* sparkles new insight into corneal dystrophies

**DOI:** 10.1038/s41431-024-01709-5

**Published:** 2024-10-11

**Authors:** Joni A. Turunen

**Affiliations:** 1 Eye Genetics Group, Folkhälsan Research Center, Biomedicum Helsinki, Helsinki, Finland; 2 Ophthalmic Genetics and Rare Eye Diseases Service, Department of Ophthalmology, University of Helsinki and Helsinki University Hospital, Helsinki, Finland

**Keywords:** Genetics research, Clinical genetics

The cornea acts as the eye’s primary tissue to bend (refract) light, being responsible for approximately 70% of the eye’s total refractive power. When light enters the eye, it first passes through the cornea, which refracts the light rays with the lens to focus them on the retina, where light is converted into neural signals for the brain to interpret as an image. The cornea must remain transparent and regularly shaped to transmit and refract light efficiently. Any opacities or irregularities in the cornea can lead to distorted vision.

Corneal dystrophies are a group of hereditary disorders limited to the cornea, most often characterized by the abnormal accumulation of material in its layers, which can lead to visual impairment. These diseases are usually bilateral, but there can be differences in the progression of the dystrophy between the eyes. In recent years, significant progress has been made in identifying the causative genes behind inherited corneal dystrophies [1]. The International Committee for the Classification of Corneal Dystrophies (IC3D) was created in 2005 to develop a classification system integrating information on phenotype, histopathology, and genetic data. Recently, the committee published the 3rd edition of the classification, including now 22 entities [2].

In this issue, Braddock et al. [3] present an autosomal dominant corneal stromal dystrophy in a pedigree with eight affected individuals in three generations. They found a very likely pathogenic variant in a new causative gene, *SPARCL1*. The affected individual had diffuse haze in the corneal stroma (the middle, thickest cellular layer of the cornea). Other corneal layers as well as other parts of the eye were unaffected, making it the possible 8th corneal stromal dystrophy. Corneal findings resemble pre-Descemet corneal dystrophy (PDCD) so far without known genetic cause [2], but the authors note that the haze was present in the anterior and mid-stroma, whereas PCCD, as its name indicates, is located in the posterior stroma, making the authors’ dystrophy likely a new entity. Braddock et al. excluded variants from all known corneal dystrophy genes and identified a segregating missense variant in the *SPARCL1* gene, which has not been previously linked to any corneal dystrophy. Interestingly, SPARCL1 stimulates the expression of decorin [4], a known gene behind congenital stromal corneal dystrophy (CSCD), making this gene functionally a plausible cause for the new dystrophy.

The stop-codon-introducing variants in the *DCN* gene result in the production of an abnormally short version of the decorin protein, which disrupts normal collagen structure and function within the corneal stroma, affecting its transparency [5]. In their paper, the authors show immunohistochemical data about the localization and expression levels of these two proteins; decorin was downregulated in the affected stroma, but *SPARCL1* was upregulated in the epithelial layer, which is not the pathologically affected layer. *DCN* is also lost together with keratocan (*KERA*), lumican (*LUM*), and epiphycan (*EPYC*) in posterior amorphous stromal dystrophy (PACD), which is characterized by stromal opacities in the first decade of life [6]. It is hoped that research will continue and show precisely how SPARCL1 protein may affect decorin and other stromal molecular components.

The variant and gene, *SPARCL1*, indeed is a possible cause for new corneal dystrophy, but because only one family is presented, we need to wait for new unrelated affected patients with pathogenic variants in the same gene to emerge. Therefore, this article is important so that inherited corneal dystrophy researchers can now examine the *SPARCL1* gene in their genetically unsolved samples, and the gene can be added to clinical gene panels. The future will show whether this finding is ultrarare or rather turns out to be a frequent cause of stromal dystrophy. Further, because the phenotype had a relatively late onset, it is interesting to see the evolving phenotypic spectrum of this corneal dystrophy.
